# The complete chloroplast genome sequence of *Veratrum nigrum* L.

**DOI:** 10.1080/23802359.2022.2050475

**Published:** 2022-03-09

**Authors:** Wen-Xiao Men, Yue-Yue Song, Che Bian, He-Fei Xue, Yan-Ping Xing, Liang Xu, Ming Xie, Ting-Guo Kang

**Affiliations:** School of Pharmacy, Liaoning University of Traditional Chinese Medicine, Dalian, China

**Keywords:** Chloroplast genome, Melanthiaceae, phylogenetic analysis, *Veratrum nigrum*

## Abstract

The complete chloroplast genome of an important medicinal plant, *Veratrum nigrum* Linnaeus, was sequenced. The entire circular genome is 151,580 bp in length, with 37.7% GC contents. The genome has a large single-copy (LSC) region with a length of 81,806 bp, a small single-copy (SSC) region with a length of 17,472 bp, and two inverted repeat regions (IRs) with a length of 26,151 bp. It harbored 131 genes, including 85 protein coding genes, 38 tRNA genes, and eight rRNA genes. Phylogenetic analysis suggested *V. nigrum* formed a monophyletic clade with relatively short genetic distance to *Veratrum oxysepalum* and *Veratrum taliense.* This study will provide theoretical basis for further study on plant genetics phylogenetic research.

*Veratrum nigrum* Linnaeus (1753), a perennial herb of Melanthiaceae, is widely distributed in the north temperate zone according to the record of Flora Reipublicae Popularis Sinicae (FRPS). It is used as Chinese herbal medicine, providing efficacy of emetic, dispelling pathogenic wind and eliminating phlegm, detoxifying and expelling parasites. Recent studies of several species in this genus showed that *Veratrum* has medicinal functions such as analgesic, anti-inflammatory, anti-fungal, anti-tumor, anti-hypertensive effect (Li et al. 2006; Augustin et al. 2015; Li et al. 2016). Bioactive substances of secondary metabolites isolated from veratrum plants are steroid alkaloids, stilbenes, and flavonoids. Other components mainly include phytosterols, coumarins, lignans, and fatty acids (Cheng and Rao 2021). Current studies analyzed the phylogenetic relationship of *Veratrum* by genome sequencing and microscopy (Han et al. 2019; Hu et al. 2020). It should be noted that *Veratrum* has both biological activity and strong toxicity. For its rational exploitation, *Veratrum* needs further research on its pharmacodynamic mechanism and systematic classification.

According to the Regulations of the People's Republic of China on Wild Plants Protection, *V. nigrum* is not in the list of national key protection of wild plants. On-site and *ex situ* protection of wild plants and scientific research on wild plants are supported in article five of the regulations. With the permission of Pharmacy College in Liaoning University of traditional Chinese Medicine in May 2018, the experimental materials were collected from the college herbal garden in China (E 121°53′14″, N 39°4′12″), and identified by professor Ting-guo Kang in this university. All operations are carried out in accordance with guidelines in Specification on Good Agriculture and Collection Practices for Medicinal Plants (GACP; Number: T/CCCMHPIE 2.1-2018). The voucher specimen and genomic DNA were deposited at the herbarium of Liaoning University of Chinese Medicine (Liang Xu 861364054@qq.com, *V*. *nigrum* number: 10162210511033LY). Total genomic DNA was extracted from fresh leaves by Magbead Plant DNA Kit (CWBIO, Jiangsu, China) and sequenced on Illumina Novaseq 6000 platform. Data editing and assembling were accomplished by NGS QC toolkit (Patel and Jain 2012) and SPAdes v3.11.0 (Bankevich et al. 2012), respectively. The protein coding sequences of chloroplast were compared with NR protein databases for protein-coding gene prediction and annotation.

The chloroplast genome length of *V. nigrum* was 151,580 bp, including a large single-copy (LSC) region with a length of 81,806 bp, a small single-copy (SSC) region with a length of 17,472 bp, and two inverted repeat regions (IRs) with a length of 26,151 bp. The genome harbored 131 genes, including 85 protein coding genes, 38 tRNA genes, and eight rRNA genes, with a GC content of 37.7%. Additionally, we found that 15 genes, including trnk-UUU, *rps*16, trnG-UCC, *atp*F, *rpo*C1, trnL-UAA, trnV-UAC, *pet*B, *pet*D, *rpl*16, *rpl*2, *ndh*B, trnI-GAU, trnA-UGC, and *ndh*A, each of which contains one intron, *clp*P and *ycf*3 genes contain two introns, and *rps*12 gene has trans splicing.

The complete chloroplast genome of 22 species of plant and the outgroup *Ginkgo biloba* were selected for phylogenetic analysis by IQ-TREE 1.6.12 software (Nguyen et al. 2015) (bootstrap value 1000) with the model K3Pu + F+R6 chosen according to the Bayesian information criterion (BIC) (Figure 1). The phylogenetic tree shows that *V. nigrum* formed a monophyletic clade with relatively short genetic distance to *V. oxysepalum* and *V. taliense*. In conclusion, the complete chloroplast genome was determined in this study, which provides theoretical foundation for further study on the phylogenetic relationship of Melanthiaceae family.

**Figure 1.**
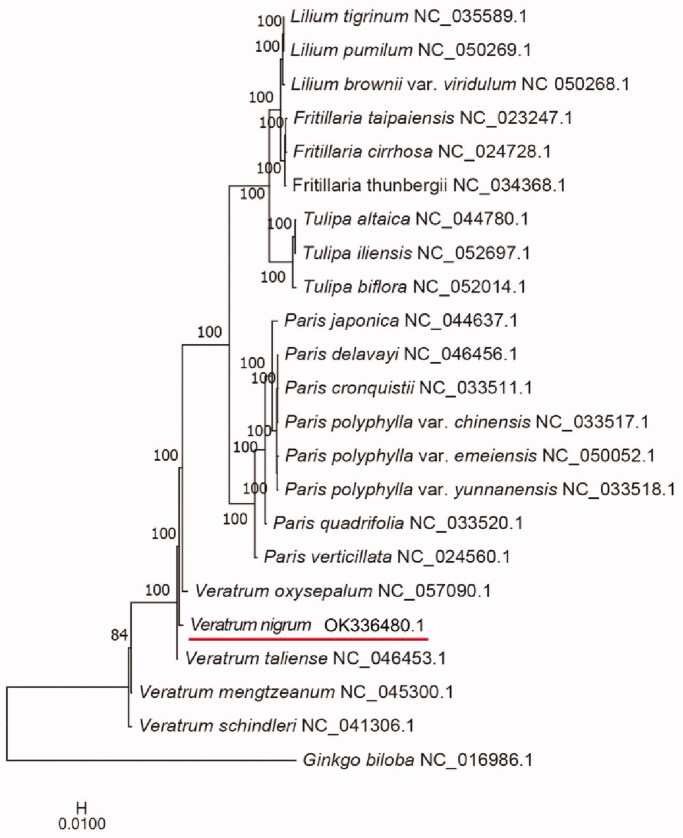
Maximum-likelihood (ML) phylogenetic tree based on the complete chloroplast genome of *V. nigrum* and 22 other species.

## Authors contributions

W.X.M. and L.X. designed the study; Y.Y.S., C.B., H.F.X., and Y.P.X. carried out the sampling and analyses; T.G.K. and M.X. were involved in validation and supervision; W.X.M. and L.X. contributed to the writing and revising.

## Data Availability

The genome sequence data that support the findings of this study are openly available in GenBank of NCBI at https://www.ncbi.nlm.nih.gov/ under the accession no. OK336480. The associated BioProject, SRA, and Bio-Sample numbers are PRJNA767490, SRX12408188, and SAMN21906506, respectively.
